# Enhancing Precision and Efficiency of Cas9-Mediated Knockin Through Combinatorial Fusions of DNA Repair Proteins

**DOI:** 10.1089/crispr.2023.0036

**Published:** 2023-10-10

**Authors:** Ryan R. Richardson, Marilyn Steyert, Saovleak N. Khim, Garrett W. Crutcher, Cheryl Brandenburg, Colin D. Robertson, Andrea J. Romanowski, Jeffrey Inen, Bekir Altas, Alexandros Poulopoulos

**Affiliations:** Department of Pharmacology and UM-MIND, University of Maryland School of Medicine, Baltimore, Maryland, USA.

## Abstract

Cas9 targets genomic loci with high specificity. For knockin with double-strand break repair, however, Cas9 often leads to unintended on-target knockout rather than intended edits. This imprecision is a barrier for direct *in vivo* editing where clonal selection is not feasible. In this study, we demonstrate a high-throughput workflow to comparatively assess on-target efficiency and precision of editing outcomes. Using this workflow, we screened combinations of donor DNA and Cas9 variants, as well as fusions to DNA repair proteins. This yielded novel high-performance double-strand break repair editing agents and combinatorial optimizations, yielding increases in knockin efficiency and precision. Cas9-RC, a novel fusion Cas9 flanked by eRad18 and CtIP^[HE]^, increased knockin performance *in vitro* and *in vivo* in the developing mouse brain. Continued comparative assessment of editing efficiency and precision with this framework will further the development of high-performance editing agents for *in vivo* knockin and future genome therapeutics.

## Introduction

CRISPR technologies have enabled the development of agents to edit the genome in naive host cells and whole organisms. The development of high-performance *in vivo* editing agents offers the potential for cost- and time-effective knockin experiments in wild-type (WT) organisms without the need for transgenesis and animal lines. The broad efficacy of Cas9 across biological systems holds further promise for knockin research in non-model organisms^1^ and future genome therapeutics in humans.^2^

A key challenge for genome editing *in vivo* is outcome precision.^3^ Existing tools offer adequate precision for *ex vivo* editing, where correctly edited cells can be selected, expanded, and validated into single-clone populations. To achieve reliable editing directly *in vivo*, where selection is typically impractical, editing agents must perform with high efficiency as well as high precision to minimize unintended on-target outcomes. Developing editing agents with high performance in both efficiency and precision remains a challenge for the field.

The Cas9 toolkit offers a variety of editing modalities, including double-strand break (DSB) repair-based editing, base editing, and reverse transcriptase-based (Prime) editing.^4^ DSB repair-based approaches are maximally versatile for knockin, allowing insertions from DNA donors that enable knocking in larger domains, such as fluorescent proteins. However, such approaches are also the most imprecise, often leading to unintended disruptions in gene function.^5^ Current DSB repair-based knockin methods produce the desired editing outcome the minority of the time, an order-of-magnitude less than unintended on-target insertions or deletions (indels).^3^

In this study, we present a high-throughput workflow to quantify editing outcomes for creating and identifying editing agents with increased performance and their optimal combinations for knockin applications. We established editing *efficiency* and *precision* as generalizable assessment metrics for comparisons of knockin performance across existing and novel agents. Using this platform, we aimed to enhance DSB repair-based editing performance by combinatorial screens of Cas9 variants, DNA donors, and new compound fusions to DNA repair protein domains. This workflow yielded Cas9-RC, a high-performance DSB repair-based editing agent with increased editing efficiency compared to Cas9 and increased precision compared to Cas9-CtIP^[HE]^. We tested Cas9-RC for its editing performance *in vivo* in the embryonic mouse brain, where it enhanced fluorescent protein knockin in some cases by *in utero* electroporation. These improvements showcase the utility of this workflow for continued development and assessment of new precision editing tools for *in vivo* knockin applications.

## Methods

### Plasmid design and construction

Mammalian expression plasmids and knockin donor template plasmids were constructed with a combination of standard cloning techniques. For gRNA expression constructs, oligos (Integrated DNA Technologies) were annealed and cloned into a custom hU6 backbone using Golden Gate Assembly (GGA).^6^ Cas9 expression constructs were assembled by a modified mMoClo system.^7^

Briefly, individual parts were cloned, BsaI adapters added, and internal sites removed by polymerase chain reaction (PCR) using KAPA HiFi HotStart DNA Polymerase with 2X Master Mix (Roche), or synthesized (Integrated DNA Technologies). Parts were subsequently assembled into expression constructs using NEB GGA Kit (BsaI-HF v2) according to the manufacturer's recommendations. Homology arms for donor templates were PCR amplified from CD1 mouse genomic DNA with adapters for GGA. gRNA binding site (GRBS) parts were generated by oligo annealing. GRBS and homology arms were assembled with knockin sequences using GGA. See Supplementary Table S1 for sequences.

### Cell line culture and transfection

BFP knockin HEK293 cells (HEL:BFP) were developed in the Corn Lab and were the kind gift of Chris Richardson.^8^ Cells were maintained at 37°C and 5% CO2 in Dulbecco's modified Eagle's medium (DMEM) media plus GlutaMax (ThermoFisher Scientific) supplemented with 10% (v/v) fetal bovine serum (FBS). Typically, 20,000–22,500 cells/cm2 were seeded onto 24-well plates the day before transfection. Cells were transiently transfected at 70–80% confluence using Polyethylenimine, Linear, MW 25000 (“PEI,” Polysciences) resuspended to 1 mg/mL in H2O at a 3:1 (v/w) PEI:DNA ratio with 250 ng DNA per plasmid (750 ng total DNA) diluted in Opti-MEM (ThermoFisher Scientific) and added dropwise to cells.

### Flow cytometry

Cells were trypsinized, pelleted, and resuspended in Dulbecco's phosphate-buffered saline (PBS) containing 0.1% FBS. At least 20,000 live cells (typically 80,000+) were analyzed using an LSRII cell analyzer with HTS (BD Biosciences). Blue fluorescent protein (BFP) and mTagBFP were measured with a 407 nm laser and a 450/50 emission filter. Green fluorescent protein (GFP) and mNeonGreen were measured with a 488 nm laser, a 505 LP mirror, and a 530/30 emission filter. mCherry was measured with a 561 nm laser, a 600 LP mirror, and a 615/25 emission filter. Data were analyzed with FlowJo v10.6.2 (Flowjo LLC). Live cells were gated by size and granularity using forward scatter (FSC-A) versus side scatter (SSC-A). Singlets were gated using SSC-A versus SSC-H (Supplementary Fig. S1). At least three biological replicates were run with internal technical duplicates or triplicates.

### Sequence analysis of knockin products

Transiently transfected HEK:BFP cells were sorted using a FACSAria II sorter (BD Biosciences). Genomic DNA was extracted from sorted cells using the Genomic DNA Clean & Concentrator kit (Zymo Research). PCR fragments were amplified using KAPA HiFi HotStart DNA Polymerase with 2X Master Mix (Roche), gel extracted with Zymoclean Gel DNA Recovery kit (Zymo Research), and submitted for Sanger sequencing (Genewiz). See Supplementary Table S1 for primer sequences. Alignment of sequencing results was performed using Benchling. Analysis of editing outcomes by decomposition of Sanger sequencing data was performed using the inference of CRISPR edits (ICE) Analysis tool v2 (Synthego) as previously described.^9^

### Animals

All animal experimental protocols were approved by the University of Maryland Baltimore Institutional Animal Care and Use Committee and complied with all relevant ethical regulations regarding animal research. Experiments were performed on outbred strain CD1 mouse pups (Charles River Laboratories). Analyses are thought to include animals of both sexes at approximately equal proportions, as no sex determination was attempted. No statistical method was used to predetermine sample size.

### Primary cell culture and cuvette electroporation

CD1 mouse pups were euthanized by decapitation on postnatal day 0. The skin was sterilized and removed from the pup's back using sterile surgical tools. Skin was placed dermis-side down on cold 0.25% Trypsin with ethylenediaminetetraacetic acid (Invitrogen) and incubated at 4°C overnight. The epidermis was separated from the dermis in a sterile hood. Dermis was minced with a razor blade and triturated in warm 10% FBS 1 × GlutaMAX DMEM using a glass pipette 10–20 times to separate individual cells.

The suspension was then transferred to a 50 mL conical tube and centrifuged at 150 g. The cell pellet was resupsended in 10% FBS 1 × Glutamax DMEM and filtered through a 100 μm cell strainer (BD Biosciences). Cells were counted using a hemocytometer and cell viability was estimated using Trypan Blue (Sigma). Approximately 4–5 × 10^6^ cells were used for each electroporation. Cells were centrifuged at 150 g and resuspended in 100 μL AMAXA nucleofection solution (Lonza) at the proper concentration and combined with 1–3 μg of desired DNA mixture in a cuvette.

The cuvette was electroporated with the AMAXA biosystems Nucelofector II (Lonza) using the manufacturer's settings for Mouse Embryonic Fibroblasts. After electroporation, the solution was immediately transferred to 12-well glass bottom plates (#1.5H; Cellvis), which were pretreated with poly-L-lysine (Sigma Aldrich) diluted 1:12 in sterile PBS the night before, containing prewarmed sterile-filtered DMEM (ThermoFisher) supplemented with 10% FBS (Gibco) and 1 × GlutaMAX (Gibco) at the desired density and incubated at 37°C/5% CO_2_. Half volume fresh medium was exchanged the next day.

### *In utero* electroporation

Electroporations of plasmid DNA were performed *in utero* on embryonic day 14.5 (E14.5) to target cortical layer II/III, as previously described.^10,11^ To increase transfection efficiency in the cortex and to target the cerebellar Purkinje cell progenitor zone at E11.5, the triple electrode *in utero* electroporation approach was utilized.^12,13^ Briefly, DNA solutions were prepared to 4 μg/μL total DNA, with 1 μg/μL of each relevant plasmid (donor, guide, Cas9, and fluorescent protein). Dams were deeply anesthetized with isoflurane under a vaporizer with thermal support (Patterson Scientific Link7 & Heat Therapy Pump HTP-1500). The abdominal area was prepared for surgery with hair removal, surgical scrub, and 70% ethanol and 10% Betadine solution.

A midline incision was made to expose the uterine horns. Using pulled (Narishige PC-100) and beveled (Narishige EG-45) glass micropipettes connected to a pneumatic aspirator, DNA solution was injected into one lateral brain ventricle for cerebral cortex electroporation on E14.5, or in the fourth ventricle for cerebellar Pukinje cell electroporation on E11.5. Around 4 × 50 ms square pulses of 35 V (NEPA21 electrokinetic platinum Tweezertrodes connected to a BTX ECM-830 electroporator) were applied to target the nascent sensorimotor areas of the cortical plate. When using the triple electrode, 6 × 50 ms square pulses at 35V was performed on E14.5 and 25V on E11.5.

Typically, four to six pups were electroporated per dame. Uterine horns were placed back inside the abdominal cavity, and monofilament nylon sutures (AngioTech) were used to close muscle and skin incisions. After term birth, electroporated mouse pups were noninvasively screened for unilateral cortical or cerebellar fluorescence using a fluorescence stereoscope (Leica MZ10f with X-Cite FIRE LED light source) and returned to their dame until postnatal day 7 (P7) or P14. When possible, to minimize interdame variation, control and experimental electroporations were performed in littermate pups from the same dame.

### Histology and immunolabeling

Tissue was prepared by intracardial perfusion with PBS and 4% paraformaldehyde. Brains were cut to 80 μm coronal sections on a vibrating microtome (Leica VT1000). Sections were immunolabeled in blocking solution consisting of 5% bovine serum albumin and 0.2% Triton X-100 in PBS for 30 min, and then incubated overnight at 4°C with primary antibodies diluted in blocking solution. Sections were washed in PBS, incubated for 3–4 h at room temperature with secondary antibodies diluted 1:400–1:1000 in blocking solution. Following PBS washes, sections were mounted on slides with Fluoromount-G Mounting Medium with 4′,6-diamidino-2-phenylindole (DAPI) (ThermoFisher Scientific). For antibodies used, see *Antibodies* tab in Supplementary Table S1.

### Microscopy and image analysis

Fluorescence images were acquired using a Nikon Ti2-E inverted microscope fitted with an automated registered linear motor stage (HLD117; Pior Scientific), a Spectra-X 7 channel LED light engine (Lumencor), and standard filter sets for DAPI, FITC, TRITC, and Cy5. Images were stitched and analyzed with NIS-Elements (Nikon) using an automated script to identify and count electroporated cells in brain sections. Knockin-positive neurons were counted manually using ImageJ (NIH) and independently by at least two blinded investigators. Five 80 μm sections, centered at the middle of the anteroposterior axis of the electroporation field and taken every other section, were analyzed per brain, with counts aggregated across sections from the same brain.

### Statistical analysis

All statistical values are presented as mean ± standard error of the mean. For experiments containing more than two conditionals or groups, statistical significance was calculated using a one-way analysis of variance (ANOVA) with Tukey's multiple comparison test, with a single pooled variance. For experiments containing two conditionals or groups, statistical significance was calculated using a two-tailed Student's *T* test. Effect size was calculated using Cohen's *d* and expressed in pooled standard deviations.^14^ Differences between conditions were judged to be significant at *p* < 0.05 (*), *p* < 0.01 (**), and *p* < 0.001 (***).

### Citation diversity statement

Toward awareness and mitigation of citation biases, we used cleanBib to assess the predicted gender and predicted racial/ethnic category of the first and last author of our cited references. The gender breakdown of our references is 11.05% woman (first)/woman (last), 9.28% man/woman, 22.18% woman/man, and 57.49% man/man. The racial/ethnic breakdown of our references is 24.39% author of color (first)/author of color (last), 13.8% white author/author of color, 31.36% author of color/white author, and 30.45% white author/white author. These analyses exclude self-citations and are subject to the caveats and limitations outlined in the cleanBib documentation.

## Results

### Quantifying editing efficiency and precision through BFP-to-GFP conversion

To quantify and compare *Streptococcus pyogenes* Cas9 and donor variant combinations on knockin performance, we used a BFP-to-GFP conversion assay previously developed using an engineered HEK293 cell line (HEK:BFP) genomically expressing BFP.^8^ In this study, we developed donor templates in a variety of formats to target the BFP sequence and convert it to GFP by introduction of the point mutation H26Y.

**FIG. 1.**
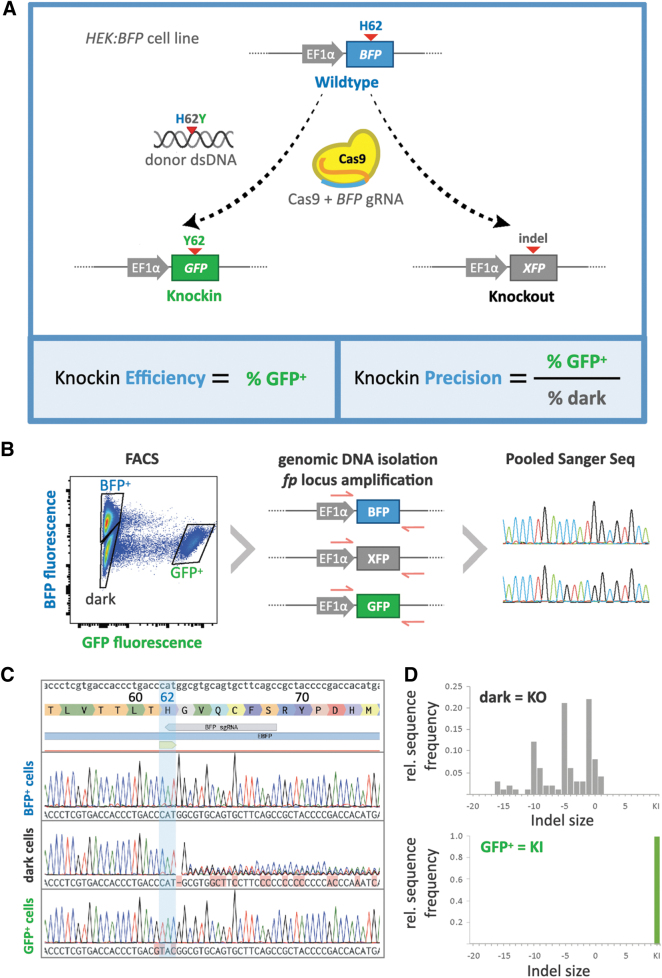
BFP-to-GFP editing as a platform to quantify knockin efficiency and precision. **(A)** Schematic of BFP-to-GFP conversion assay screen. Genome editing agents are tested by transfecting *HEK:BFP* cells, a HEK293 knockin cell line that genomically expresses BFP driven by an EF1α promoter. Successful editing knocks in the H62Y mutation into the *BFP* locus, thus producing GFP. Precisely edited cells will change from blue to green, while imprecise edits on the *BFP* locus will largely result in indel knockouts and disrupt fluorescence, turning those cells from blue to dark. Quantification of knockin efficiency is calculated by the proportion of GFP+ cells over total, and knockin precision is calculated by the proportion of GFP+ cells over dark cells. **(B)** Schematic outlining sorting and sequencing of cells treated with agents to edit the *BFP* locus. Fluorescence plot shows FACS isolation of distinct cell populations following treatment for H62Y editing. GFP fluorescence is shown on the x-axis (log), and BFP fluorescence on the y-axis (log). Blue, green, and dark collection gates are indicated. Genotyping of the three collected populations was performed by PCR of the *BFP* locus from genomic DNA, followed by Sanger sequencing of amplified fragment pools. **(C)** Alignment of Sanger sequencing reads from BFP^+^, dark, and GFP^+^ cell populations to the reference *BFP* locus sequence (top), showing wild-type, knockout (indel), and knockin genotypes, respectively. **(D)** Editing outcomes (indels and knockin frequency) were quantified by decomposition of Sanger sequencing reads using the ICE algorithm and plotted on relative histograms binned by indel size. GFP^+^ cells represent true knockins, and over 90% of dark cells contain indels predicted to cause knockout. BFP, blue fluorescent protein; EF1α, translation elongation factor 1A; FACS, fluorescent activated cell sorting; GFP, green fluorescent protein; HEK, human embryonic kidney; ICE, inference of CRISPR edits; PCR, polymerase chain reaction.

Fluorescence was used as a surrogate for editing outcomes following transfection of these cells with Cas9 or Cas9 variants, gRNA targeting the *BFP* locus, and H26Y donor DNA formats. Precise H26Y knockin would cause fluorescence to shift from BFP to GFP, while on-target indels by error-prone repair would result in unintended knockout and loss of fluorescence (Fig. 1A). By quantifying the proportions of BFP^+^, GFP^+^, and dark (BFP^−^/GFP^−^) cells, we can assess knockin efficiency (% of GFP^+^ to BFP^+^ cells) and knockin precision (ratio of % GFP^+^ to % dark cells) as internally controlled ratios across editing agents.

To validate our workflow and confirm that fluorescence readouts correspond to predicted genotypes in HEK:BFP cells, we used fluorescent activated cell sorting (FACS) to sort and collect the three phenotypic cell populations (BFP^+^, GFP^+^, and dark) that emerge following treatment with BFP/H26Y editing agents. Genomic DNA was extracted from sorted cell populations and used as template in PCR with primers flanking the region targeted by the BFP gRNA within the *BFP* locus, thereby amplifying all alleles regardless of on-target editing outcome. Pooled PCR amplicons from each sorted cell population were analyzed by Sanger sequencing (Fig. 1B).

As expected, the genotype of the BFP^+^ population matched that of the WT *BFP* sequence. The dark population exhibited a complex mixture of sequence results in the vicinity of the BFP gRNA cleavage site, representing unintended on-target edits due to imprecise repair. Sequencing decomposition using ICE (ref 9) and TIDE (Tracking of Indels by DEcomposition)^15^ on amplicons from the dark sorted population revealed a predominance of deleterious indels (79% frameshift vs. 11% in-frame), in line with a loss of fluorescence due to knockout. Finally, the GFP^+^ population exhibited a single genotype containing the desired H26Y point mutation knockin from the donor template (Fig. 1C, D). The sequencing results matched the fluorescence surrogates, thus validating this platform for high-throughput comparative screening of knockin agents.

### Combinatorial screening of editing agents for enhanced performance

**FIG. 2.**
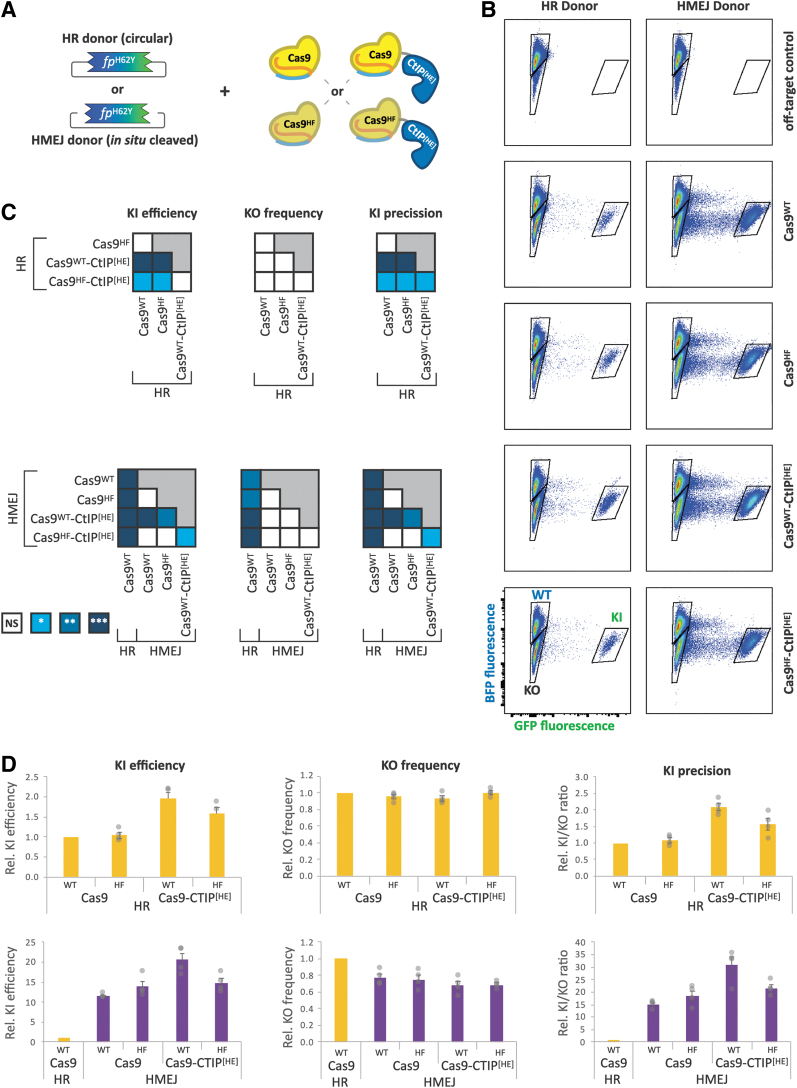
Cas9-CtIP^[HE]^ fusion and HMEJ donors additively improve knockin precision. **(A)** Schema illustrating the combinatorial parameters of editing agents: dsDNA donor template HR or HMEJ variants in combination with Cas9 WT or HiFi variants, alone or fused to CtIP^[HE]^. **(B)** Flow cytometry plots of *HEK:BFP* cells 7 days after transient transfection with gRNA, Cas9, and donor plasmids. GFP fluorescence is shown on the x-axis (log), and BFP fluorescence on the y-axis (log). Quantification gates are indicated on the plots for BFP^+^ (WT), GFP^+^ (KI), and dark (KO) cells. **(D)** Quantification of flow data representing normalized knockin efficiency (%GFP+), knockout frequency (% dark), and knockin precision. Cas9 variants with HR (top row, yellow) or HMEJ (bottom row purple) donors were measured. Mean values from individual experiments (*n* ≥ 3) were normalized to those of the Cas9 WT with HR donor condition and presented as the mean ± SEM. **(C)** Heat maps displaying statistical significance of pairwise comparisons of editing agent performance, calculated using one-way ANOVA with Tukey's multiple comparison test and single pooled variance (*p < 0.05; **p < 0.01; ***p < 0.001). Cas9-CtIP^[HE]^ fusion with HMEJ donor outperforms Cas9 with HR donor in knockin precision by over 30-fold. ANOVA, analysis of variance; HMEJ, homology-mediated end joining; HR, homologous recombination; KI, knockin; KO, knockout; SEM, standard error of the mean; WT, wild type.

We sought to identify elements of DSB repair knockin agents that in combination offer improved efficiency and precision of editing. We investigated a matrix of combinations of three factors, which have individually been shown to enhance knockin performance: (1) WT *S. pyogenes* Cas9 (“WT” Cas9) versus a High-Fidelity (“HF”) Cas9 sequence variant (Cas9-HF)^16,17^; (2) Cas9 variants alone versus fusions with the “HDR Enhancing” (HE) N-terminal fragment (1–296) of the DNA repair protein CtIP,^18,19^ which we refer to as CtIP^[HE]^; and (3) circular DNA donor predicted to favor homologous recombination (HR) versus *in situ* linearized DNA donor predicted to favor homology-mediated end joining (HMEJ)^20,21^ (Fig. 2A).

Both knockin donors were provided on plasmids with the knockin sequence flanked by ∼800 bp homology arms, the length of which did not significantly affect results within a range of 500–1500 bp (data not shown). The HMEJ donor differed from the HR donor by the insertion of GRBS flanking the homology arms, which are cleaved by Cas9 to create linear dsDNA donors in cells. The orientation of the GRBSs did not have significant effects on editing performance (Supplementary Fig. S2).

Transfection of HEK:BFP cells with Cas9 variant, donor template, and the *BFP* gRNA led to the emergence of both knockin (GFP^+^) and knockout (dark) populations for all combinations of donors and Cas9 variants, while transfection with off-target control gRNA resulted in no GFP^+^ cell (Fig. 2B). Among Cas9 variants tested, Cas9^WT^-CtIP^[HE]^ performed with a roughly twofold improvement in knockin efficiency compared to Cas9^WT^, regardless of dsDNA donor variant, consistent with previous reports.^19,22,23^ Interestingly, CtIP^[HE]^ fusion did not significantly improve efficiency and precision for single-strand oligodeoxynucleotide DNA donors (Supplementary Fig. S3). Cas9^HF^ variants were generally not significantly different from Cas9^WT^, although Cas9^HF^-CtIP^[HE]^ showed a 1.6-fold improvement in knockin efficiency, specifically with the HR donor (Fig. 2D). Knockout rates did not significantly differ among the Cas9 variants (Fig. 2C), and thus, the knockin-to-knockout ratios (knockin precision) mirror the differences seen in knockin efficiency (Fig. 2D).

In contrast to the Cas9 variants, donor architecture impacted knockin efficiency as well as knockout rates. Across all four Cas9 variants, the HMEJ donor showed a 9- to 13-fold increase in knockin efficiency compared to the corresponding HR combination (Supplementary Fig. S4). Of the combinations of Cas9 and donors tested, Cas9^WT^-CtIP^[HE]^ in conjunction with the HMEJ donor resulted in the highest rate of knockin, 24-fold higher than Cas9^WT^ with the HR donor.

Regardless of Cas9 variant, the HMEJ donor showed a 22–30% reduction in gene disruption, which, together with the improved knockin efficiency, enhanced the precision by about 15-fold relative to the HR donor template permutations (Supplementary Fig. S2). Interestingly, these data suggest the independent and additive contributions of both CtIP^[HE]^ fusion (twofold) and the *in situ* cleaved HMEJ donors (∼11-fold) to knockin efficiency. Taken together, these results demonstrate that combinatorial optimization of both Cas9 and donor DNA can significantly shift the balance away from error-prone repair, with the combination of Cas9^WT^-CtIP^[HE]^ (henceforth “Cas9-CtIP^[HE]^”) and HMEJ donors giving the highest efficiency and precision in HEK cells.

### Compound fusions on Cas9 improve editing performance

**FIG. 3.**
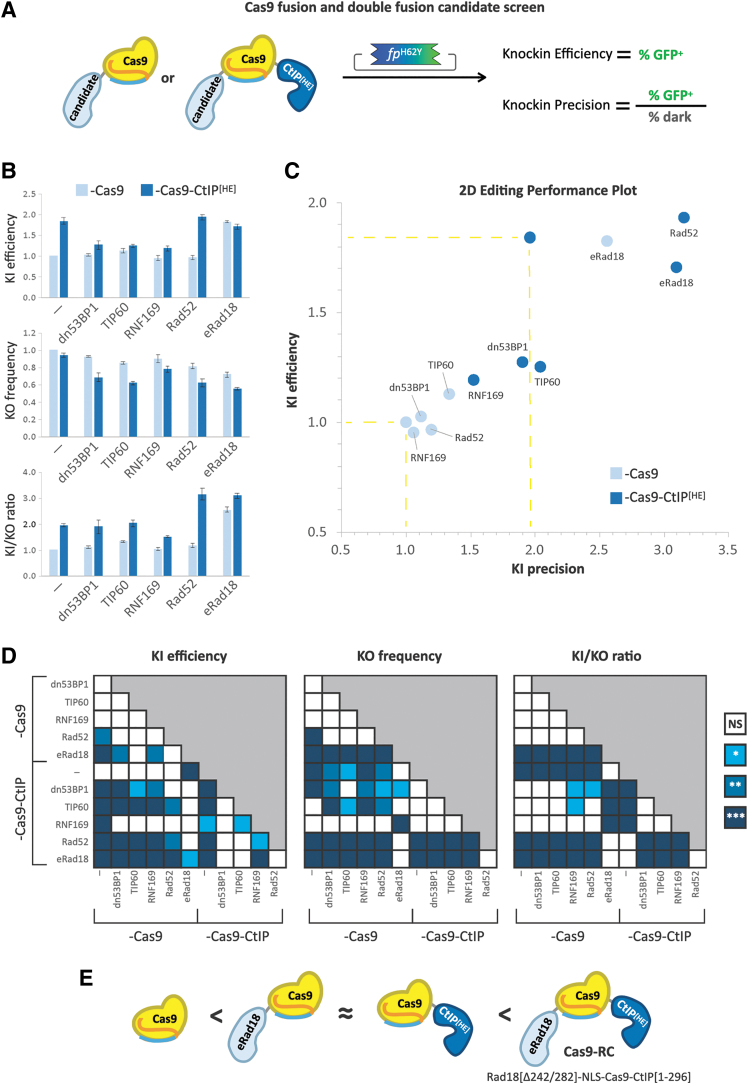
Iterative screening of novel Cas9 fusions and compound fusions with DNA repair domains for increased editing performance. **(A)** Schema of the fusion screen. Candidate DNA repair protein domains are fused N-terminally to either Cas9 alone or Cas9-CtIP^[HE]^ and together with HMEJ donor assayed by BFP-to-GFP for knockin efficiency and precision. **(B)** Bar graphs showing quantification of relative knockin efficiency, knockout frequency, and knockin precision for Cas9 fusion (light blue) or Cas9-CtIP^[HE]^ compound fusion (dark blue) with the listed DNA repair protein domains. Values from individual experiments (*n* ≥ 3) were normalized to Cas9 only and presented as the mean ± SEM. Data of individual replicate experiments shown in Supplementary Figure S5. **(C)** 2D editing performance plot comparing relative knockin efficiency and precision of Cas9 fusions (light blue) or Cas9-CtIP^[HE]^ compound fusions (dark blue). Points with yellow dashed lines projecting to the axes correspond to Cas9 alone (normalization reference) and Cas9-CtIP^[HE]^. **(D)** Heat map matrices showing statistical significance of data in **(B)** and **(C)** calculated using a one-way ANOVA with Tukey's multiple comparison test and pooled variance. Differences between conditions were judged to be significant at *p* < 0.05 (*), *p* < 0.01 (**), and *p* < 0.001 (***). **(E)** Schema illustrating comparative performance outcomes: novel fusion eRad18-Cas9 has equivalent performance to Cas9-CtIP^[HE]^. Novel compound fusion eRad18-Cas9-CtIP^[HE]^, henceforth named Cas9-RC, outperforms single fusions.

Several groups have independently demonstrated that modulation of DNA repair pathways is an effective way to improve knockin efficiency and precision.^19,23,24^ To build on the results of the 3-factor screening, highlighting WT Cas9, CtIP^[HE]^ fusion, and HMEJ donor as the best performing combination, we iterated the BFP-to-GFP screening platform with constant HMEJ donor and evaluated the impact of five candidate DNA repair protein domains (dn53BP1,^24–26^ TIP60,^26,27^ RNF169,^25^ Rad52,^23–30^ and eRad18^27^) on editing efficacy and precision when fused N-terminally to Cas9 or Cas9-CtIP^[HE]^ (Fig. 3A).

In the absence of CtIP^[HE]^, only eRad18 fusion to Cas9 significantly improved knockin efficacy, increasing it by 1.8-fold above Cas9 alone, similar to Cas9-CtIP^[HE]^. With the compound fusions, while addition of dn53BP1, TIP60, or RNF169 to Cas9-CtIP^[HE]^ appeared to abrogate the effect of CtIP^[HE]^ on knockin efficacy, fusion of Rad52 or eRad18 did not have a detrimental impact on efficiency (Fig. 3B, D).

Regarding unintended on-target knockout, dn53BP1-, TIP60-, and RNF169-fused Cas9 did not significantly differ from the Cas9-only control. Rad52 and eRad18 fusion, however, showed 18% and 28% reductions in knockout frequency, respectively (Fig. 3B, D). Interestingly, compound fusion of each of the five DNA repair proteins with CtIP^[HE]^ led to significant reductions in the knockout rate, with eRad18, Rad52, and TIP60 showing the most pronounced decreases (45%, 38%, and 38%, respectively). Despite these reductions in knockout rates, only Rad52 and eRad18 led to significant improvements in overall knockin precision. Without CtIP^[HE]^, eRad18 demonstrated a 2.5-fold increase in the knockin-to-knockout ratio, while combination of either Rad52 or eRad18 with CtIP^[HE]^ led to a 3.1-fold increase in precision relative to Cas9 alone (Fig. 3B, D). These results show that specific combinations of DNA repair domains fused to flank Cas9 can function together to improve both the efficiency and precision of editing.

We elected to move forward with the smallest of these compound fusions, Cas9^WT^ flanked by eRad18 and CtIP^[HE]^, which we call “Cas9-RC.” In addition to C-terminal fusion of truncated human CtIP^[HE]^, Cas9-RC harbors an N-terminal fusion of a deletion variant of human Rad18 protein, eRad18, which lacks the SAP domain between residues 242 and 282 (Δ242/282), for a total of 2,172 residues. eRad18 was shown to enhance homology-dependent repair (HDR) when independently co-expressed with Cas9 by suppressing imprecise end-joining repair pathways.^27^

Cas9-RC works with both HMEJ and HDR donor templates. However, because eRad18 suppresses recruitment of 53BP1, which is required for both nonhomologous end joining and microhomology-mediated end joining (MMEJ),^31^ we did not test Cas9-RC with MMEJ donors, which would be predicted to perform poorly. Cas9-RC expression plasmids and test knockin plasmids have been made available through Addgene (Cas9-RC with GFP #207404 or mScarlet #207405; test KI-HMEJ #207407, 207408; Supplementary Table S1).

### Cas9-RC increases knockin *in vivo*

The combinatorial screening and iterative optimizations of Cas9 agents yielded Cas9-RC, which showed increases in knockin performance in cultured cell lines. Aiming to develop precision knockin agents for direct editing *in vivo*, we sought to assess the knockin efficacy of Cas9-RC with an HMEJ donor in a mouse model.

**FIG. 4.**
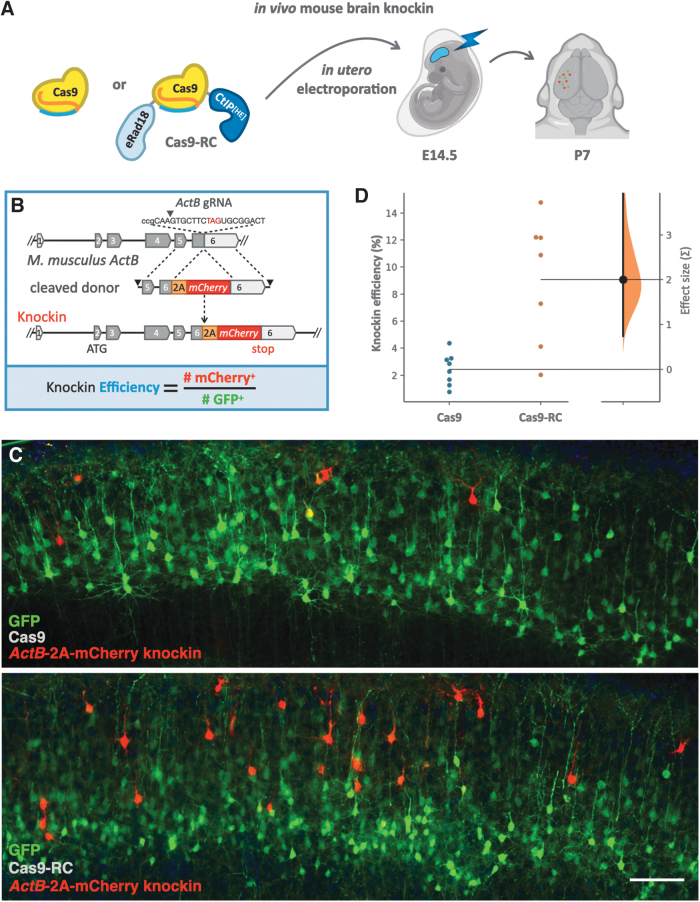
Cas9-RC enhances knockin efficiency *in vivo*. **(A)** Schema of the Cas9 and Cas9-RC agents compared for *in vivo* editing using *in utero* electroporation in the fetal mouse brain on embryonic day (E) 14.5, and analysis in the cerebral cortex on postnatal day (P) 7. **(B)** Gene editing donor template DNA targets the endogenous *ActB* locus to insert mCherry downstream of the β-actin coding sequence separated by a 2A. Knockin efficiency was quantified as the number of mCherry^+^ (knockin) neurons over the number of GFP^+^ (electroporated) neurons. **(C)** Representative fluorescence images of the cerebral cortex on P7 with electroporated neurons receiving Cas9 or Cas9-RC. Images show plasmid GFP (green) and genomic *ActB*-2A-mCherry (red) expression. Scale bar 100 μm. **(D)** Swarm plots showing quantification of *in vivo* knockin efficiency for Cas9 versus Cas9-RC and effect size estimation. Points show means from each brain and are plotted on the left axis for both groups indicating knockin efficiency. The effect size on knockin efficiency of Cas9-RC versus Cas9 is plotted as a distribution on a floating axis on the right, indicating standard deviations (Σ). The effect size estimated by unpaired Cohen's *d* between Cas9 and Cas9-RC is 2.0 Σ (black dot), indicating a large effect size. The 95% confidence interval is 0.739 to 3.97 (vertical error bar). The *p* value is 0.0022.

To test the knockin efficiency of Cas9-RC *in vivo*, we used *in utero* plasmid electroporation^10^ in the embryonic mouse brain^32^ (Fig. 4A), targeting integration of a 2A mCherry cassette at the 3′ end of the endogenous β-Actin (*ActB*) locus (Fig. 4B). A combination of four plasmids containing Cas9 or Cas9-RC, HMEJ donor with the 2A mCherry knockin, *ActB* gRNA, and a GFP transfection marker was electroporated into embryonic day 14.5 (E14.5) wild-type mice targeting progenitors of projection neurons of sensorimotor cortex. When assessing knockin efficiency in neurons on postnatal day 7 (P7), electroporation of Cas9-RC led to an increase in mCherry^+^ knockin neurons compared to Cas9 (Fig. 4C).

*In vivo* knockin efficiency was calculated by comparing the number of mCherry^+^ knockin neurons to the number of GFP^+^ electroporated neurons (Fig. 4B). Electroporation of Cas9 resulted in an *in vivo* knockin efficiency of 2.4% (±0.44%), while Cas9-RC yielded a 3.7-fold increase in performance, averaging 9% (±1.7%) *in vivo* knockin efficiency (Fig. 4C, D). These results demonstrate that Cas9-RC can display increased knockin performance over Cas9 both *in vitro* and *in vivo*.

### Fluorescent protein knockin applications with Cas9-RC

We went on to use Cas9-RC plasmids and HMEJ donors in knockin applications of three different fluorescent proteins onto three different loci in three different cell types.

**FIG. 5.**
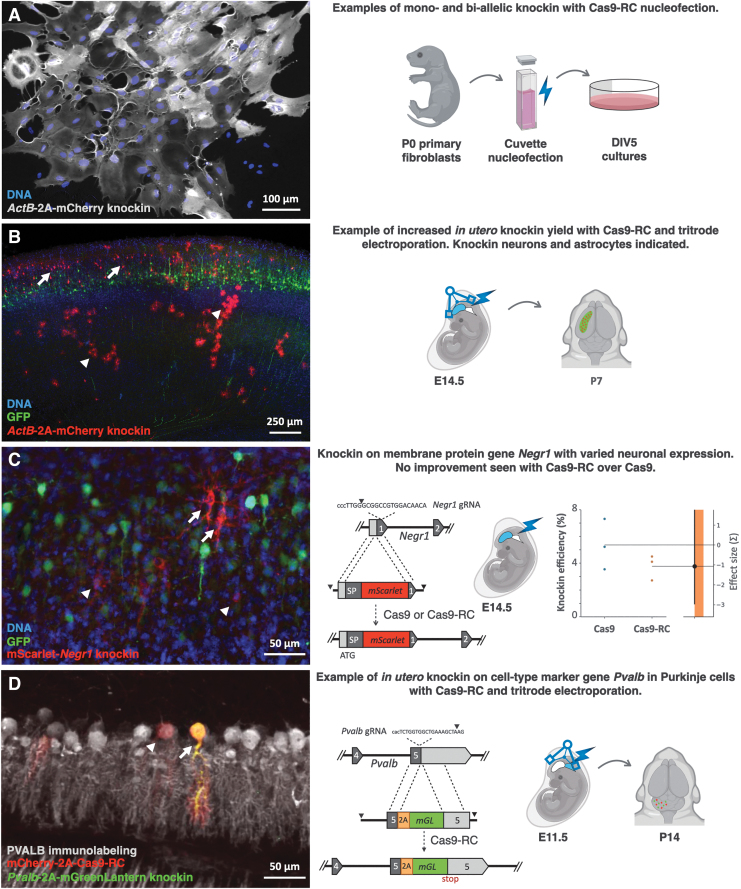
Knockin applications with Cas9-RC. **(A)** Primary fibroblasts from WT newborn mice were cuvette electroporated (nucleofected, schema on the right) with Cas9-RC plasmid and *ActB*-2A-mCherry donor DNA. Fibroblasts show two intensities of mCherry fluorescence (red) from the mouse β-Actin locus, suggesting mCherry knockin on one (dim) or both (bright) *ActB* alleles. **(B)** Cortical neurons (arrows) and astrocytes (arrowheads) are knocked in with Cas9-RC plasmid and *ActB*-2A-mCherry donor DNA *in utero* as in Figure 4, but with supercoiled plasmids and high-yield tritrode *in utero* electroporation. Increasing the efficiency of large plasmid electroporation is a significant practical determinant of knockin yield. **(C)** Knockin to fuse mScarlet extracellularly onto Negr1, a GPI-anchored membrane protein expressed at variable levels in neurons. Cas9-RC and mScarlet-*Negr1* HMEJ donor DNA were *in utero* electroporated into E14.5 mouse brain. Knockin cells displayed variable levels of mScarlet fluorescence (red) with a wide range of high (arrows) and low (arrowheads) expressing neurons. Quantification (right panel as in Fig. 4D) of mScarlet-positive cells over GFP electroporated cells (green) showed no significant difference when using Cas9-RC over Cas9. **(D)** Knockin of mGreenLantern into the *Pvab* locus, a marker gene for cerebellar Purkinje cells. Cas9-RC (red) and *Pvalb*-2A-mGreenLantern donor DNA were delivered with high-yield tritrode electroporation into the fourth brain ventricle in E11.5 embryos (schema on the right). Endogenous PVALB protein was immunolabeled (gray). Electroporated knockin positive (arrow) and knockin negative (arrowhead) Purkinje cells can be seen in P14 cerebellum. In gene schemata, exon numbers, SP, ATG start codon, and stop codons are indicated. Color labels and scale bars as indicated. SP, signal peptide.

We extracted primary mouse fibroblasts from P0 mice and *ex vivo* electroporated Cas9-RC, *ActB*-2A-mCherry donor, and *ActB* gRNA plasmids (same as used in Fig. 4) using cuvette electroporation (nucleofection). Knockin cells displayed mCherry fluorescence with two distinct intensities, as would be characteristic of cells expressing mCherry from one or both *ActB* alleles (Fig. 5A). Biallelic knockin cells offer experimental advantages because of increased fluorescent signal, and because biallelic expression would indicate that both alleles remain functional after editing. Due to the high incidence of indels with conventional HDR-based editing, cells that display monoallelic knockin are likely to have loss-of-function indels on the second allele, potentially causing unintended haploinsuficiency phenotypes. We have not quantified the frequency of biallelic knockin; however, the increased knockin and decreased knockout rates observed with Cas9-RC compared to Cas9 *in vitro* (Fig. 3B) may increase the chances of obtaining biallelic knockins.

One of the limitations of Cas9-RC efficiency *in vivo* is the large size of the expression construct, which drastically reduces electroporation efficiency. We repeated Cas9-RC *in utero* electroporations with Cas9-RC, *ActB*-2A-mCherry knockin constructs in the brain using a triple electrode^12,13^ (tritrode) and highly supercoiled plasmid DNA, both of which increased the efficiency of electroporation. We were able to obtain considerable numbers of knockin cells, including both neurons and astrocytes, using Cas9-RC with supercoiled plasmids and the tritrode method (Fig. 5B). Our experience with Cas9-RC indicates that with steps to increase electroporation of large plasmids, significant knockin efficiency can be achieved for applications where high knockin cell numbers are critical.

We used the same knockin approach to fuse the fluorescent protein mScarlet onto the N-terminus of the GPI-linked membrane protein neuronal growth regulator 1 (Negr1), a protein with variable expression in the mouse brain.^33^ *Negr1* knockin differs from actin knockin in that not all knockin cells will express Negr1,^34^ and some cells express it at levels below our detection threshold.

We went on to quantify mScarlet fluorescence compared to GFP electroporation marker to assess Cas9-RC knockin efficiency on the *Negr1* locus compared to Cas9, but found no significant difference (Fig. 5C). This may indicate that increases of Cas9-RC performance *in vivo* may be locus specific. Alternatively, it may indicate that the lower electroporation efficiency of Cas9-RC *in vivo* compared to the smaller Cas9 may negate the positive effects of Cas9-RC in loci with low knockin yield. This result indicates that Cas9-RC use over Cas9 should be confirmed for each locus individually.

Finally, we used Cas9-RC with tritrode electroporation and supercoiled constructs to target Purkinje cells in the mouse embryonic cerebellum, as a case to examine a difficult to transduce cell type that we were not able to knock in with Cas9. We constructed HMEJ donor and gRNA constructs to knock in the fluorescent protein mGreenLantern downstream of the *Pvalb* locus, a cell-type marker expressed by parvalbumin+ Purkinje cells. While efficiency was low, we consistently detected sparse knockin parvalbumin+ Purkinje cells (Fig. 5D). These results suggest that Cas9-RC may have advantages for knockin in cells that are difficult to target.

## Discussion

In this study, we sought to develop high-performance knockin tools by exploring combinations of DNA donor templates, variants of Cas9, and fusion of DNA repair protein domains. We identified novel Cas9 fusions and donor combinations that resulted in knockin with significantly improved metrics of editing efficiency and precision, *in vitro* and *in vivo*.

Our work builds upon an established reporter cell line as a standardized pipeline to optimize tools for precision genome editing. The BFP-to-GFP screening platform^8^ in HEK cells provides a high-throughput quantitative readout of the efficiency of correctly edited cells, while also reporting on the frequency of incorrectly edited cells. By simultaneously evaluating knockin and knockout rates, we identified combinations that optimized both efficiency (overall knockin rate) and precision (knockin rate vs. knockout rate). Knockin of a fluorescence cassette into the highly expressed β-Actin locus similarly enabled direct quantification of efficiency for large inserts at an endogenous gene locus *in vivo*.

Quantification at the variably expressed locus *Negr1* did not show increases in efficiency of Cas9-RC over Cas9, possibly due to the increased size of Cas9-RC, resulting in the targeting of fewer cells. Knockin on a highly expressed cell-type-specific locus (parvalbumin) allowed us to demonstrate the use of Cas9-RC on Purkinje cells, a highly differentiated cell type. The variability between loci when using Cas9-RC *in vivo* is intriguing. It may represent locus-specific parameters that determine which repair pathways are engaged,^35,36^ and thus effect repair outcomes, changing the frequency of knockin at different loci. Systematic use of Cas9-RC and other repair-pathway biasing agents across different loci may provide further information regarding the source of locus-dependent editing performance.

We propose editing *efficiency* and *precision* as generalizable performance metrics for comparing genome editing agents. The ratiometric nature of these values makes them versatile enough to be applied in a variety of biological systems, regardless of whether the experimental outputs are sequences (i.e., Fig. 1C), surrogate cellular markers (i.e., Fig. 2), or even function. These metrics incorporate all elements of the editing agent, including formulation (e.g., plasmid, RNP), editing modality (e.g., DSB repair, base editing, Prime editing), and delivery system (e.g., lipids, viral, nanoparticles), all of which may affect both outcomes.

Efficiency and precision can be used as readouts when assessing individual components for holistic performance optimization, as we have done in this study. They can additionally be useful as common metrics to compare performance across distinct editing modalities, for example, DSB repair versus Prime editing. By simultaneously assessing efficiency and precision across a variety of knockin tools, optimal agents can be selected based on the experimental need. For example, with *ex vivo* editing, one might prioritize efficiency if there are facile methods for *post hoc* selection of properly edited cells, whereas precision may be prioritized for contexts where edited cells cannot be selected, such as with *in vivo* editing.

Using this dual metric performance assessment, our study developed the high-performance DSB repair editor Cas9-RC (Supplementary Discussion). When paired with HMEJ donor templates, Cas9-RC outperformed Cas9 by over 30-fold in human cells and showed potential for threefold increases in the mouse brain, although not at all loci tested. As DSB repair through Cas9-RC enables high performance for large genomic edits, such as fluorescent protein knockin, it complements parallel developments in base editors and Prime editing, which offer high performance, but are limited to smaller edits. Ultimately, a diverse toolkit of precision editors will be useful to broaden the scope of *in vivo* editing applications. As presented in our study, having standardized platforms for quantitative comparison of new tools and novel combinations will further support efforts toward precision *in vivo* editing for both basic research and the development of future human therapeutics.

## Conclusion

Fusion of Cas9 to DNA repair protein domains can produce synergistic enhancements of knockin performance. Iterative high-throughput screening based on fluorescent protein conversion is an effective platform to assess knockin efficiency and precision for developing new editing agents. Cas9-RC is a new DSB repair genome editor demonstrating enhanced knockin performance *in vitro* and *in vivo*.

## Supplementary Material

Supplemental data

Supplemental data

Supplemental data

Supplemental data

Supplemental data

Supplemental data

Supplemental data

## Supplementary Material

Supplementary Discussion
