# Supplementary material for: Enhancing Precision and Efficiency of Cas9-Mediated Knockin Through Combinatorial Fusions of DNA Repair Proteins
Source: CRISPR J. 2023 Oct 10;6(5):447–61. doi: 10.1089/crispr.2023.0036 (PMC10611978; doi:10.1089/crispr.2023.0036)
Supplement: Supplemental data [file Suppl_FigureS1.docx]

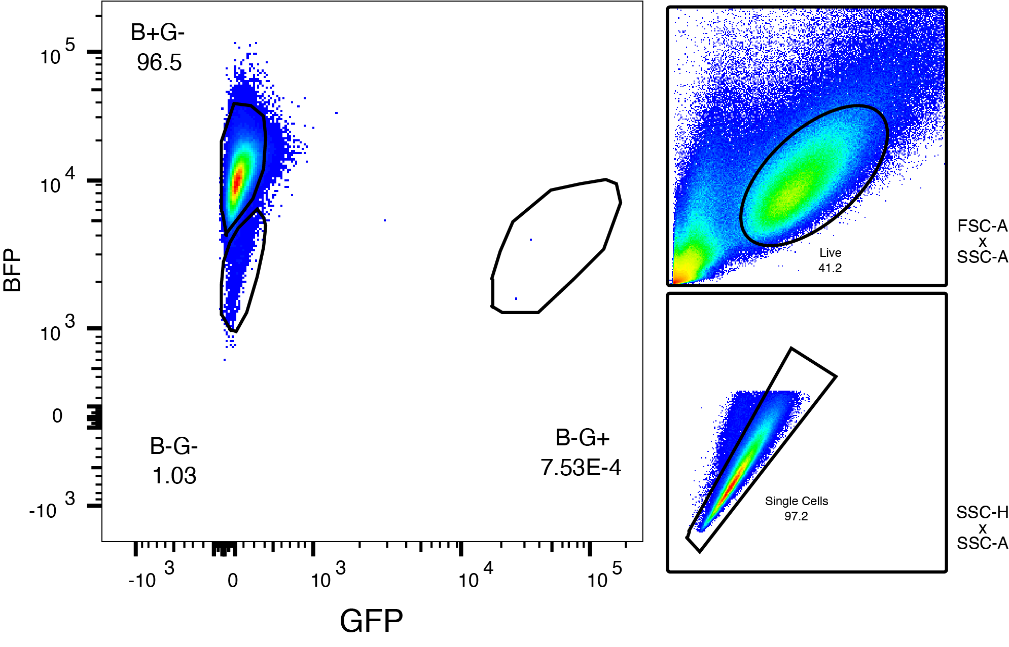


**Supplementary Fig. S1**. Gating strategy for flow cytometry analysis of editing in *HEK:BFP* cells. Live cells were first gated by size and granularity using FSC-A vs SSC-A and then singlets were gated using SSC-A vs SSC-H.
