# Supplementary material for: Enhancing Precision and Efficiency of Cas9-Mediated Knockin Through Combinatorial Fusions of DNA Repair Proteins
Source: CRISPR J. 2023 Oct 10;6(5):447–61. doi: 10.1089/crispr.2023.0036 (PMC10611978; doi:10.1089/crispr.2023.0036)
Supplement: Supplemental data [file Suppl_FigureS2.docx]

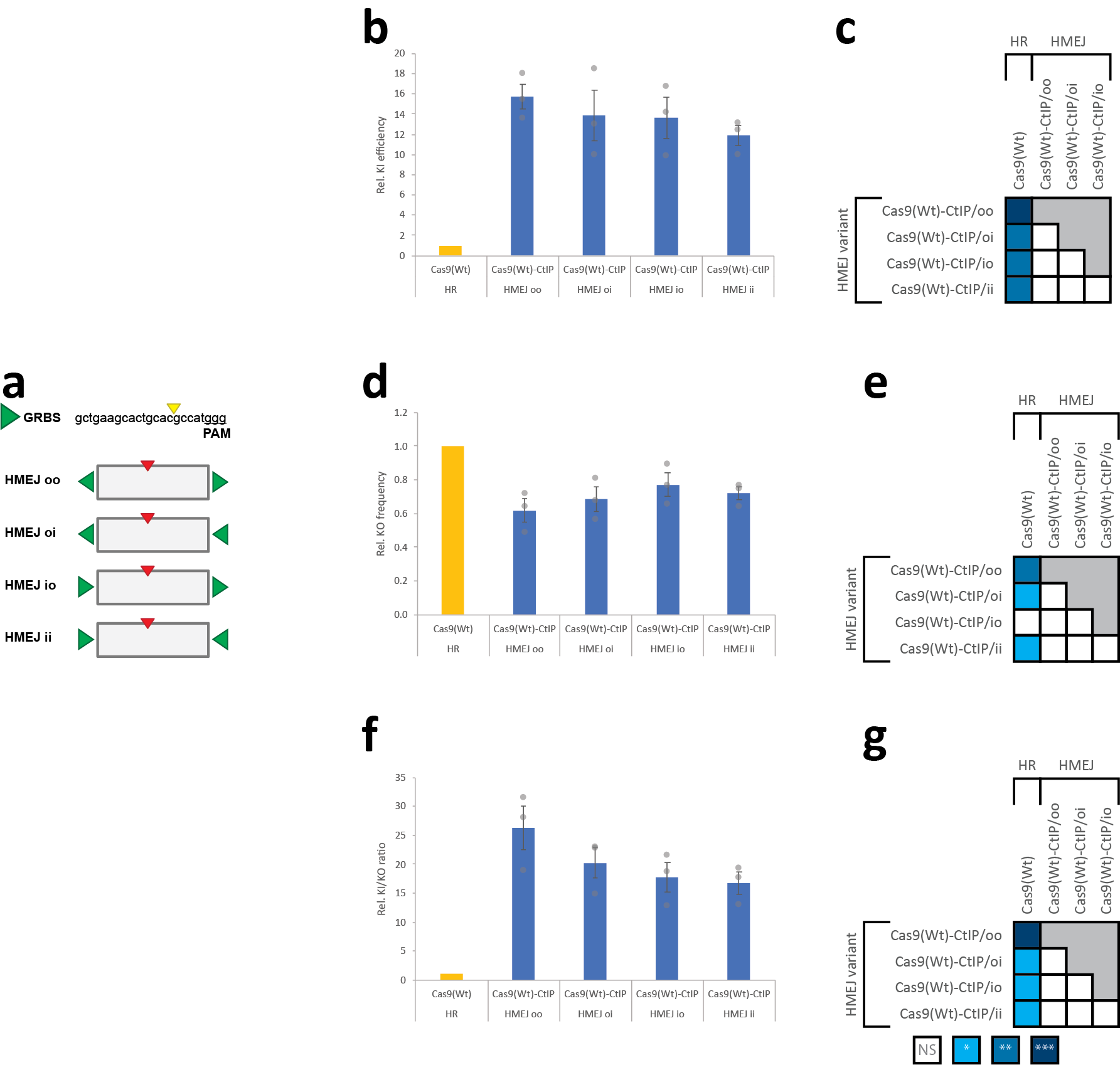


**Supplementary Fig. S2**. GRBS orientation does not impact efficiency or precision for HMEJ donors. **(a)** Schematic depicting asymmetry of BFP gRNA target sequence (GRBS) and HMEJ donors with all permutations of GRBS orientation. **(b,d,f)** Quantification of flow cytometry data from *HEK:BFP* cells 7 days after transient transfection indicating **(b)** KI efficiency (% GFP^+^) **(d)** KO efficiency (% dark) and **(f)** KI precission (KI/KO ratio) for HMEJ variants and Cas9(Wt)-CtIP^[HE]^. Values from individual experiments (n=3) were normalized to the Cas9(Wt)/HR donor condition and presented as the mean ± SEM. **(c,e,g)** Statistical significance was calculated using a one-way ANOVA with Tukey’s multiple comparison test, with a single pooled variance (*, P < 0.05; **, P < 0.01; ***, P < 0.001).
