# Supplementary material for: Enhancing Precision and Efficiency of Cas9-Mediated Knockin Through Combinatorial Fusions of DNA Repair Proteins
Source: CRISPR J. 2023 Oct 10;6(5):447–61. doi: 10.1089/crispr.2023.0036 (PMC10611978; doi:10.1089/crispr.2023.0036)
Supplement: Supplemental data [file Suppl_FigureS3.docx]

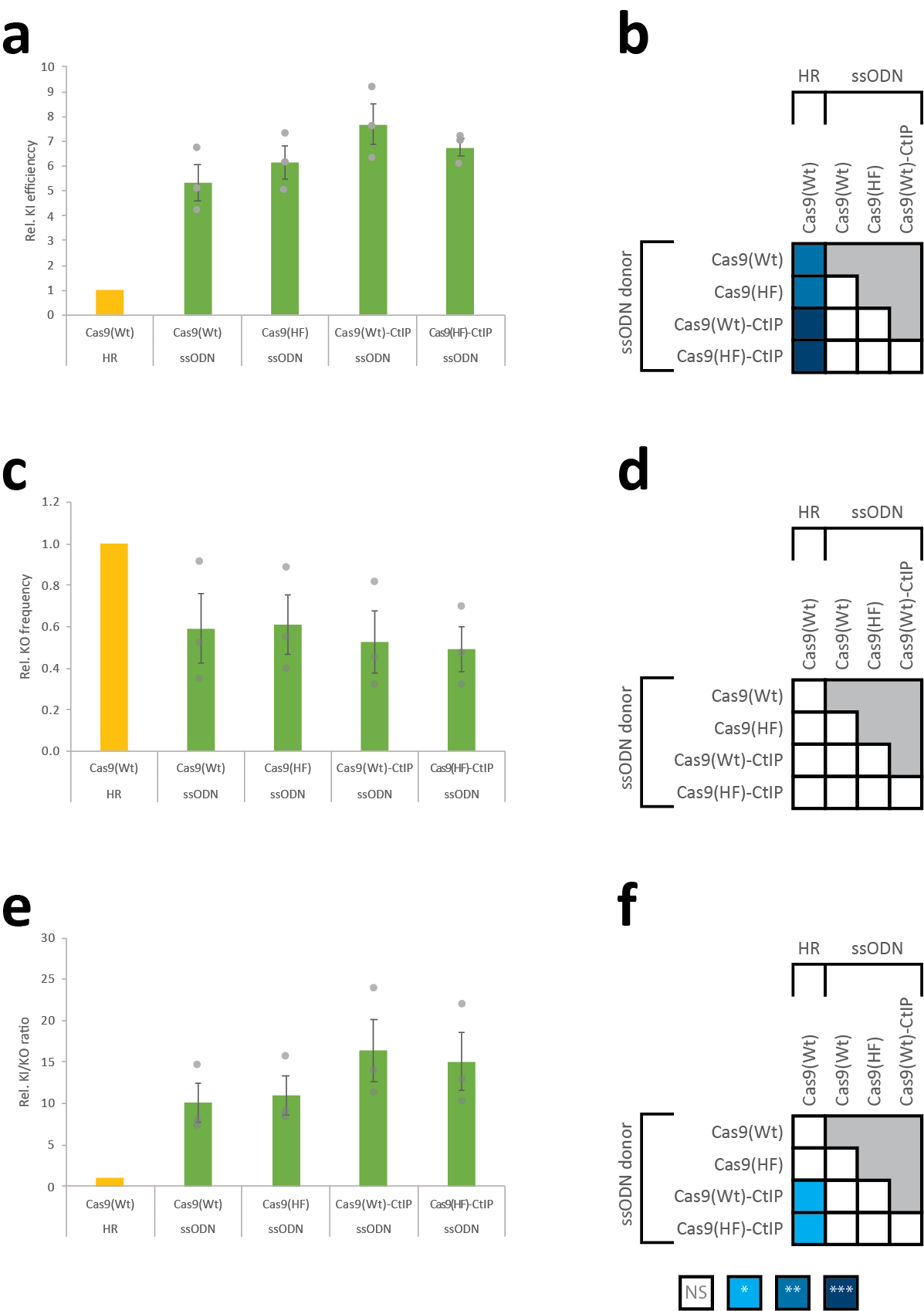


**Supplementary Fig. S3**. CtIP^[HE]^ fusion does not significantly improve efficiency and precision for ssODN donors. **(a,c,e)** Quantification of flow cytometry data from *HEK:BFP* cells 7 days after transient transfection indicating **(b)** KI efficiency (% GFP^+^) **(d)** KO efficiency (%BFP^-^) and **(f)** KI/KO ratio for Cas9 variants with a ssODN donor. Values from individual experiments (n=3) were normalized to the Cas9(Wt)/HR donor condition and presented as the mean ± SEM. **(b,d,f)** Statistical significance was calculated using a one-way ANOVA with Tukey’s multiple comparison test, with a single pooled variance (*, P < 0.05; **, P < 0.01; ***, P < 0.001).
