# Supplementary material for: Enhancing Precision and Efficiency of Cas9-Mediated Knockin Through Combinatorial Fusions of DNA Repair Proteins
Source: CRISPR J. 2023 Oct 10;6(5):447–61. doi: 10.1089/crispr.2023.0036 (PMC10611978; doi:10.1089/crispr.2023.0036)
Supplement: Supplemental data [file Suppl_FigureS4.docx]

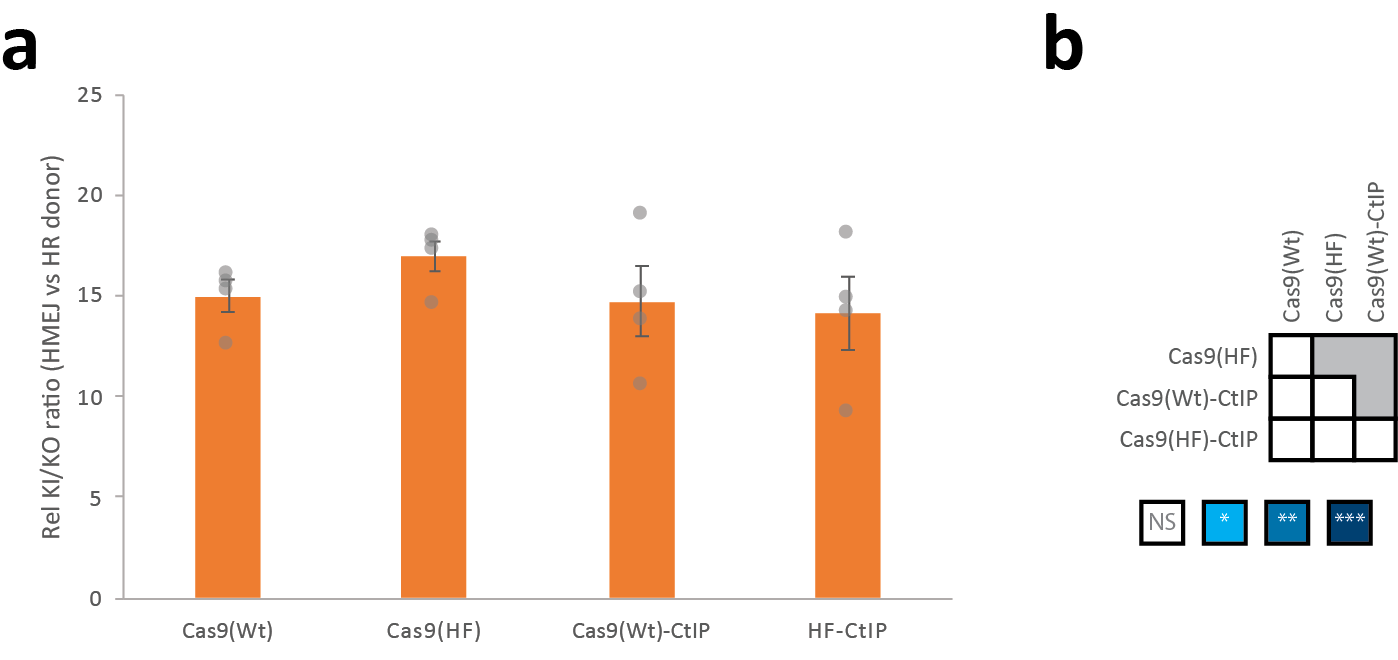


**Supplementary Fig. S4**. HMEJ donor independently improves precision across Cas9 variants. **(a)** Data related to Fig. 2 were analyzed to compare differences in precision between HR and HMEJ donors for the same Cas9 variant. **(b)** Statistical significance was calculated using a one-way ANOVA with Tukey’s multiple comparison test, with a single pooled variance. Differences between conditions were judged to be significant at P < 0.05 (*), P < 0.01 (**), and P < 0.001 (***). HMEJ outperforms HR donors by approximately 15-fold equivalently in all Cas9 iterations tested.
