# Supplementary material for: Enhancing Precision and Efficiency of Cas9-Mediated Knockin Through Combinatorial Fusions of DNA Repair Proteins
Source: CRISPR J. 2023 Oct 10;6(5):447–61. doi: 10.1089/crispr.2023.0036 (PMC10611978; doi:10.1089/crispr.2023.0036)
Supplement: Supplemental data [file Suppl_FigureS5.docx]

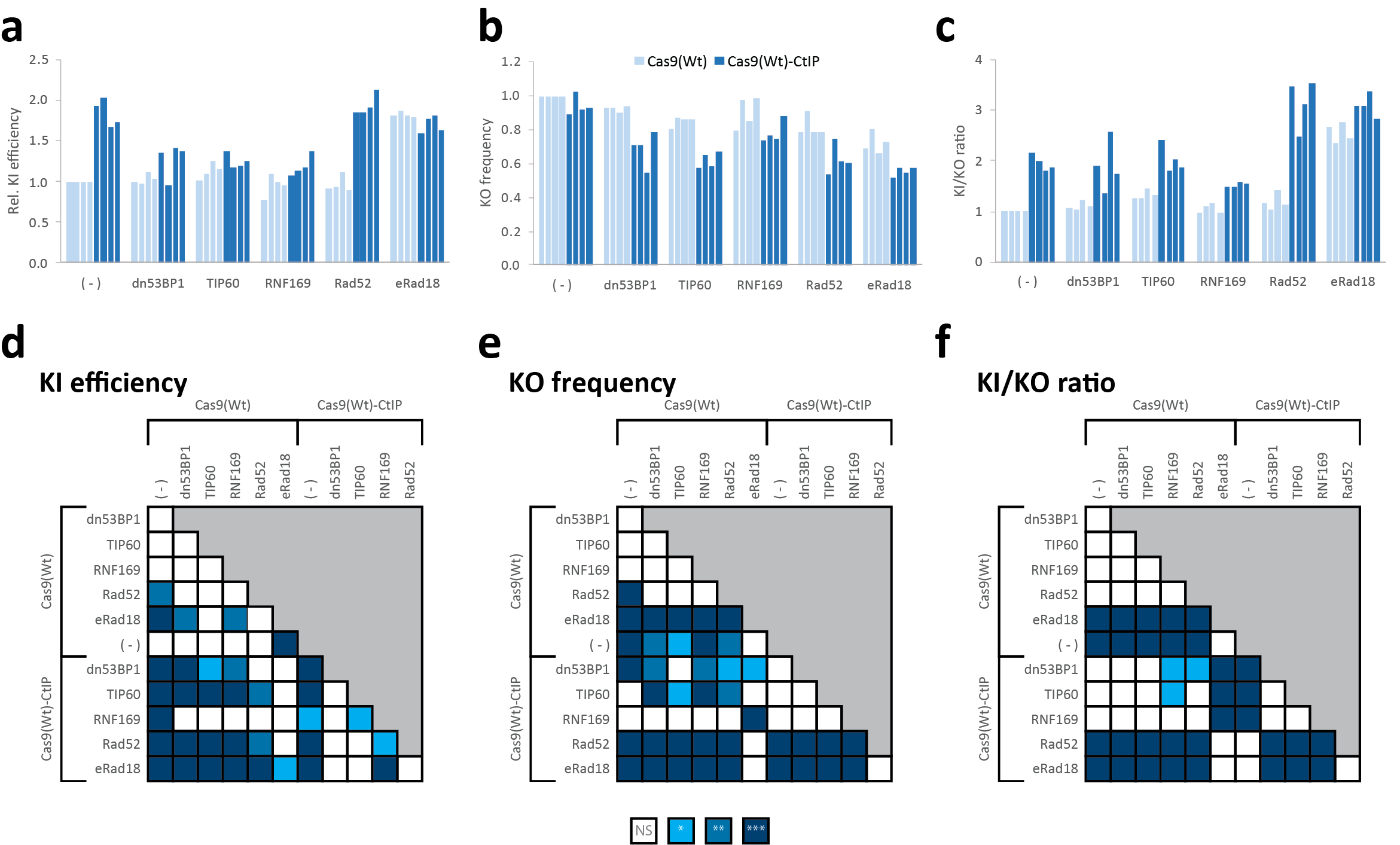


**Supplementary Fig. S5**. Biological replicate data and statistical analysis related to Fig. 3. **(a-c)** Quantification of flow cytometry data for biological replicates from HEK:BFP cells 7 days after transient transfection indicating **(a)** KI efficiency (% GFP^+^) **(b)** KO frequency (% dark) and **(c)** KI precision (KI/KO ratio) for Cas9 variants with HMEJ donor. **(d-f)** Heatmap matrices showing statistical significance calculated using a one-way ANOVA with Tukey’s multiple comparison test and pooled variance (also shown in Fig. 3). Differences between conditions were judged to be significant at P < 0.05 (*), P < 0.01 (**), and P < 0.001 (***).
